# Intrathoracic accessory spleen causing severe dysphagia: a rare cause of mediastinal compression

**DOI:** 10.1093/gastro/goaf102

**Published:** 2025-11-24

**Authors:** Florent Porez, Vincent Thomas De Montpreville, Maëlle Oger, Dominique Fabre

**Affiliations:** Department of Thoracic Surgery, Hôpital Marie Lannelongue, Groupe Hospitalier Paris Saint Joseph, INSERM UMR_S 999, Université Paris Saclay, Le Plessis-Robinson, France; Department of Anatomopatholoy, Hôpital Marie Lannelongue, Groupe Hospitalier Paris Saint Joseph, INSERM UMR_S 999, Université Paris Saclay, Le Plessis-Robinson, France; Department of Anatomopatholoy, Hôpital Marie Lannelongue, Groupe Hospitalier Paris Saint Joseph, INSERM UMR_S 999, Université Paris Saclay, Le Plessis-Robinson, France; Department of Thoracic Surgery, Hôpital Marie Lannelongue, Groupe Hospitalier Paris Saint Joseph, INSERM UMR_S 999, Université Paris Saclay, Le Plessis-Robinson, France

## Introduction

**Figure 1.**
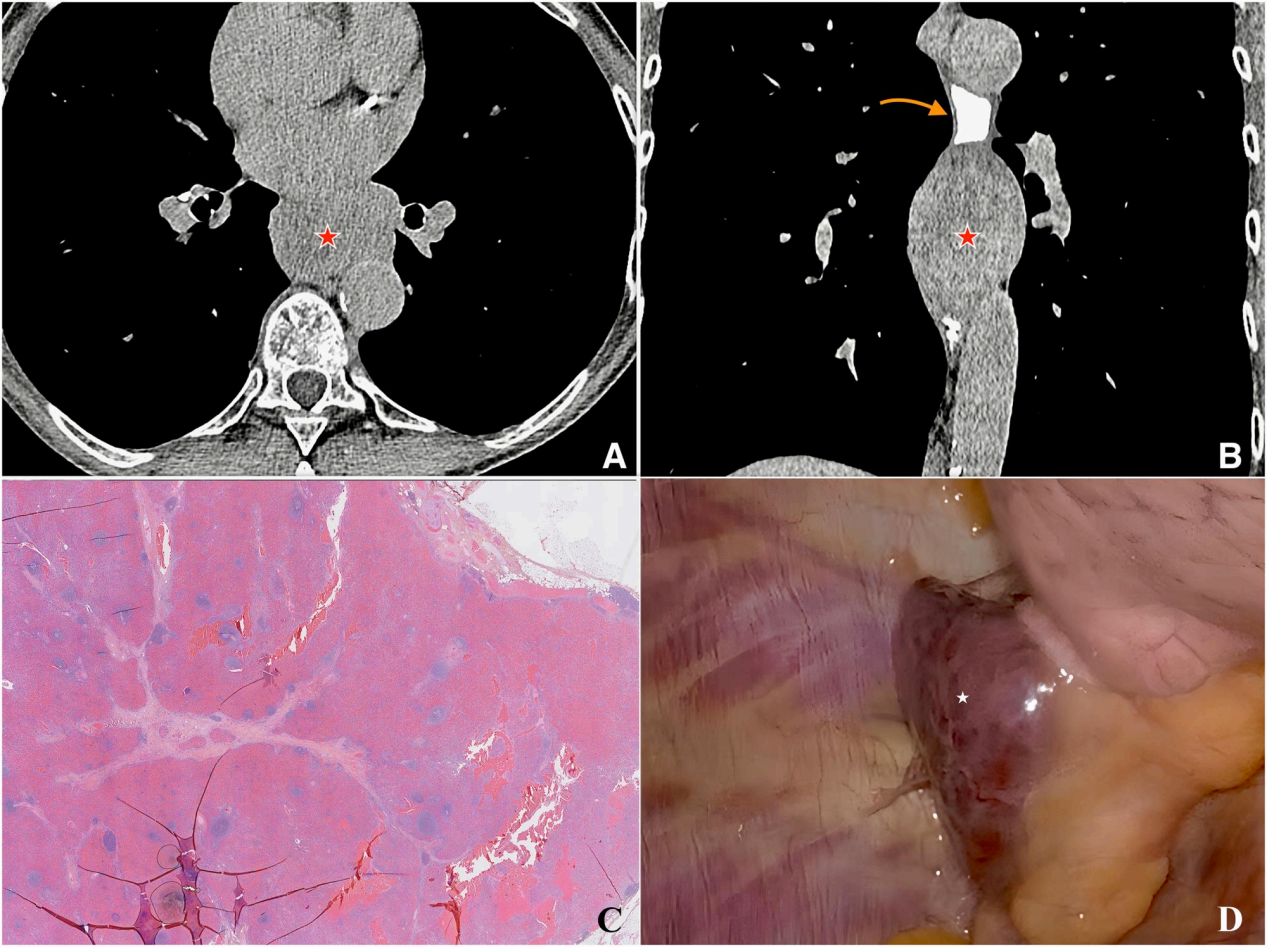
Radiologic, histologic, and intraoperative findings of a case with intrathoracic accessory spleen. (A, B) Axial and coronal computed tomography scans with digestive tract opacification showing an intrathoracic accessory spleen ( star) causing contrast stagnation and extrinsic esophageal compression (red arrow). (C) Histological section demonstrating encapsulated splenic tissue traversed by thin fibrous septa, with lymphoid follicles (white pulp) and cords of Billroth with venous sinusoids (red pulp) (hematoxylin–eosin stain, original magnification ×100, scale bar = 200 µm). (D) Intraoperative view during video-assisted thoracotomy showing the encapsulated, vascularized intrathoracic spleen (white star) after complete mobilization.

Intrathoracic accessory spleen is an exceptionally rare finding that is usually discovered incidentally and is rarely symptomatic [1–3]. This case is unique because the ectopic splenic tissue caused severe dysphagia through extrinsic esophageal compression—a presentation almost never reported (Figure 1).

It highlights the importance of considering non-gastrointestinal causes of dysphagia and the value of multimodal imaging and multidisciplinary evaluation in atypical clinical scenarios.

## Case report

A 75-year-old woman presenting with chronic, severe dysphagia prompted an in-depth gastroenterological evaluation. Although the initial esophageal endoscopy did not reveal any intrinsic abnormalities, further imaging investigations were essential to uncover the cause of her symptoms. Written informed consent for publication was obtained from the patient.

## Clinical presentation and endoscopic findings

The patient experienced progressive dysphagia that significantly affected her quality of life. Despite a comprehensive esophageal endoscopy—a critical tool in evaluating swallowing disorders—no intrinsic lesions were identified. This negative result underscored the need to explore extrinsic factors, such as compression from nearby structures, as a potential source of her symptoms.

## Imaging workup

Computed tomography (CT) with digestive tract opacification revealed an intrathoracic, retrocardiac mass that measured 45 mm in width and 80 mm in height. The mass was responsible for stagnation of the esophageal contrast and extrinsic compression of the esophagus.

A positron emission tomography scan revealed that the mass showed no metabolic activity, suggesting a benign lesion rather than a malignant process.

Initial findings from magnetic resonance imaging (MRI) were suggestive of a leiomyoma—a benign tumor known to cause mediastinal compression.

## Gastroenterological correlation

For the gastroenterologist, this case highlights the importance of considering extrinsic compressions in the differential diagnosis of dysphagia, particularly when initial endoscopic evaluations yield negative findings. The unusual presentation of an ectopic accessory spleen in the mediastinum illustrates that symptoms of dysphagia may occasionally arise from non-gastrointestinal tissues that are anatomically adjacent to the digestive tract.

## Therapeutic management

Given the patient’s debilitating symptoms and the impact on her quality of life, a minimally invasive intervention was chosen. The patient underwent a video-assisted thoracotomy to excise the mass, with careful ligation of the feeding vessels while preserving the integrity of the esophagus. The resolution of dysphagia and the absence of postoperative complications confirmed the effectiveness of this therapeutic approach.

## Discussion and teaching points for gastroenterologists

This case offers several important lessons:

- Differential diagnosis in dysphagia: When endoscopic evaluations are negative, advanced imaging is crucial to identify the extrinsic causes of the esophageal compression.
- Multidisciplinary approach: Collaboration among gastroenterologists, radiologists, and thoracic surgeons is vital for diagnosing and managing atypical cases.
- Role of complementary imaging: CT, positron emission tomography, and MRI are invaluable in differentiating benign from malignant lesions, thereby guiding appropriate therapeutic decisions.
- Embryological insight: Knowledge of embryological variations, such as the presence of an accessory splenic tissue, is essential, as these anomalies can influence gastrointestinal symptoms in unexpected ways.

## Conclusions

This rare case demonstrates that severe dysphagia may be a manifestation of extrinsic esophageal compression by an unusual mediastinal mass—specifically, an intrathoracic accessory spleen. For gastroenterologists, it is critical to consider non-gastrointestinal causes when endoscopic findings are inconclusive and to utilize complementary imaging techniques to refine the diagnostic process. A multidisciplinary approach remains key to achieving optimal patient outcomes in such atypical clinical scenarios.
